# A Comparison of Clinical Outcomes in Rotator Cuff Re-Tear Patients Who Had Either an Arthroscopic Primary Repair or Arthroscopic Patch Augmentation for Large-to-Massive Rotator Cuff Tears

**DOI:** 10.3390/diagnostics13111961

**Published:** 2023-06-04

**Authors:** Ki-Tae Kim, Gwan-Ho Kim, Dong-Heon Cha, Jae-Hoo Lee, Yong-Beom Lee

**Affiliations:** Department of Orthopedic Surgery, Hallym University Sacred Heart Hospital, Hallym University College of Medicine, Anyang 14068, Republic of Korea; kimkt8399@naver.com (K.-T.K.); kghmno@naver.com (G.-H.K.); cha92922@naver.com (D.-H.C.); holleewho@gmail.com (J.-H.L.)

**Keywords:** allograft, patch, revision, rotator cuff, shoulder arthroscopy

## Abstract

Background and Purpose: Despite the prevalent incidence of re-tear following rotator cuff repair, there is a notable lack of comparative studies investigating the outcomes between patients with re-tear who underwent primary repair versus those who received patch augmentation for large-to-massive tears. We assessed clinical outcomes of these techniques through a retrospective, randomized controlled trial. Methods: 134 patients diagnosed with large-to-massive rotator cuff tears from 2018 to 2021 underwent surgery; 65 had primary repair and 69 had patch augmentation. A total of 31 patients with re-tears were included, split into two groups; Group A (primary repair, 12 patients) and Group B (patch augmentation, 19 patients). Outcomes were evaluated using several clinical scales and MRI imaging. Results: Most clinical scores improved postoperatively in both groups. No significant difference in clinical outcomes was observed between groups, except for pain visual analog scale (P-VAS) scores. P-VAS scores showed greater decrease in the patch-augmentation group, a statistically significant difference. Conclusions: for large-to-massive rotator cuff tears, patch augmentation led to greater decreases in pain than primary repair, despite similar radiographic and clinical results. Greater tuberosity coverage of the supraspinatus tendon footprint may impact P-VAS scores.

## 1. Introduction

There are many methods for repairing large-to-massive rotator cuff tears (RCTs), including primary repair, partial repair, the margin convergence technique, medialized repair, and patch augmentation [1]. A healed tendon–bone interface should be the primary goal of rotator cuff repair, and the long-term outcome is determined by how well the tendon–bone interface is maintained [2].

Several studies reported that the re-tear rate is as high as 34–94% when primary suturing is performed in cases of large-to-massive RCTs [3]. A common cause of rotator cuff repair failure leading to re-tear is excessive tension in the suture. Re-tear can also be the result of reduced healing ability caused by decreased blood flow on the degenerated rotator cuff. The number of vascular bundles in the torn rotator cuff decreases with the degree of tendon degeneration [4]. There is a gradual decrease in the number of blood vessels as the tear size increases; as a result, the recovery process is typically more complete in smaller tears [5].

Acellular dermal matrix allograft augmentation is one possible means of addressing these problems. Using this method, the defect between the torn tendon and the greater tuberosity (GT) can be bridged when the rotator cuff is immobilized. It may also be considered when partial repair failed to restore the cuff to its footprint anatomically. Patch augmentation promotes mechanical strength and valuable biological properties by serving as a scaffold for cellular and vascular ingrowth, and collagen formation, during the reconstruction of large-to-massive rotator cuff defects [6]. Nevertheless, re-tears were also reported in patients who underwent arthroscopic patch augmentation for large-to-massive RCTs.

It is well-known that the re-tear rate of patch augmentation is lower than that of primary repair [6]. However, as far as we know, there were no studies on whether patch augmentation leads to better clinical results than primary repair, even though re-tears occur among patients in both groups. The purpose of this study was to investigate the effectiveness in clinical and functional outcomes of patch augmentation in re-tear-state shoulders in a retrospective, randomized, and controlled trial. We hypothesized that re-tear patients who underwent surgical repair augmented with an allograft dermal matrix patch would enjoy better clinical and functional outcomes, such as lower levels of pain and an improved range of motion, than re-tear patients who underwent primary rotator cuff repair.

## 2. Methods

### 2.1. Patient Selection

The study protocol was approved by the Institutional Review Board of Hallym University Sacred-Heart Hospital (No. HALLYM2020-07-034-002, on 19 January 2021), and informed consent was obtained from all patients. Data were retrospectively collected from our database and retrospectively reviewed as a single-center study. Between January 2018 and December 2021, 134 patients with large-to-massive RCTs, for whom nonoperative treatment had failed, underwent arthroscopic surgery. A total of 65 patients underwent an arthroscopic primary rotator cuff repair, and 79 patients underwent an arthroscopic rotator cuff repair with patch augmentation.

The inclusion criteria for patients in this study were: (1) a large (3–5 cm in width) or massive (>5 cm in width) rotator cuff tear [7] confirmed by preoperative magnetic resonance imaging (MRI), with pain and functional disability refractory to conservative treatment; (2) a minimum follow-up period of 6 months after surgery; and (3) re-tear on last follow-up MRI. The exclusion criteria were: (1) follow-up data not available for a minimum of 6 months after the surgical procedure; (2) a history of surgical procedures on the affected shoulder; and (3) re-operation. Following exclusion, a total of 31 patients were included in the study: 12 who underwent arthroscopic primary repair (Group A) and 19 who underwent arthroscopic repair with patch augmentation (Group B).

### 2.2. Clinical Assessments

Clinical assessments included the pain visual analog scale (P-VAS) score (range, 0 to 10), the simple shoulder test (SST), the University of California Los Angeles (UCLA) shoulder scale, the American shoulder and elbow surgeons shoulder (ASES) score, the constant shoulder score (CSS), and an assessment of the active range of motion (AROM). The aforementioned scales (SST, UCLA Shoulder Scale, ASES Score, CSS) can be interpreted such that higher scores indicate better clinical outcomes. A goniometer was used to measure the active range of motion of the shoulder to the point of pain. This included 3 movements: forward elevation (FE); external rotation (ER) with the arm at the side; and internal rotation (IR) at the back. Internal rotation was determined by measuring the highest spinal segment that the patient was able to reach with their thumb. For ease of statistical analysis of IR, the spinal segments were converted into numbers. Internal rotation was expressed as the highest vertebral level that the tip of the thumb could reach. The vertebrae were numbered in series, from below the sacrum (0) to the fourth thoracic vertebra (14) [8]. An independent examiner who was unaware of the group assignations performed the preoperative and last-follow-up clinical assessments, including shoulder scores and the active range of motion.

### 2.3. MRI Examinations

All patients participating in this study underwent shoulder MRI before and after surgery. The last follow-up MRI to check for re-tear was evaluated only if it took place at least 6 months after surgery [9]. RCT was diagnosed by the presence of liquid-like high signal intensity on a T2-weighted image containing the entire tendon substance, and by part of the rotator cuff being invisible. The same method was applied when diagnosing re-tears on MRIs after surgery [10]. The extent of retraction of the torn supraspinatus tendon was determined as the maximum medial-to-lateral length on the coronal oblique T2-weighted images, as described by Davidson et al. [11].

The extent of fatty infiltration was assessed using MRI re-adaptation of the Goutallier fatty muscle degeneration criteria (Table 1) [12]. The most useful MRI image for classification of fatty infiltration is the first lateral T1 sagittal image in which the scapula is “Y-shaped”, i.e., the scapular spine can be seen in contact with the scapular body, defining a Y bone image [13]. We assessed fatty infiltration of all 3 muscles (supraspinatus, infraspinatus, and subscapularis) on the pre-operative Y bone image. The GFDI is then calculated by adding up the grades for each of the five muscles and dividing by the number of muscles evaluated. For example, if the supraspinatus is graded 2, the infraspinatus 1, the subscapularis 0, the GFDI would be (2 + 1 + 0)/3 = 1.

**Figure 1:**
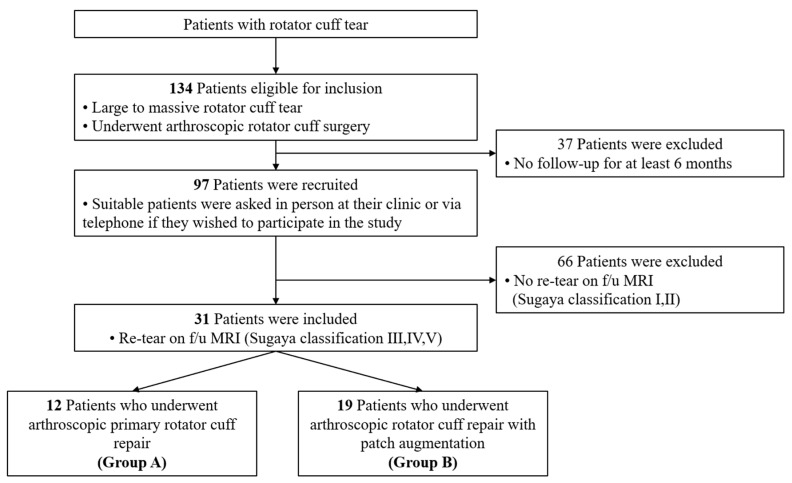
Flow chart of study participant recruitment. Recruitment criteria and the selection process are illustrated. MRI—magnetic resonance imaging. f/u—follow up.

Tendon integrity was assessed on postoperative MRIs according to the Sugaya criteria (Table 2) [14]. Grades III, IV, and V of the Sugaya categories were classified as re-tear [15,16]. For all patients, the re-tear rate was 32.0% (31 of 97 patients); re-tear rates were 18.5% (12 of 65 patients) for Group A, and 24.1% (19 of 79 patients) for Group B, respectively (Figure 1).

All pre- and postoperative MRI measurements were measured by one orthopedic surgeon.

### 2.4. Surgical Procedures

All surgical procedures were conducted by a single surgeon with over 20 years of experience in a single institution. All procedures were performed with the patient in a beach-chair position under general anesthesia. Equipment required for the procedure included a standard 30° arthroscope, three 10 mm cannulae, and suture-passing devices. Suture anchors utilized included standard triple-loaded 5.5 mm screw-in suture anchors and 5.5 mm knotless suture anchors.

Three standard shoulder arthroscopic portals (posterior, lateral, and anterolateral) were used. A posterior portal was established 1 cm medial and 2 cm distal to the posterolateral corner of the acromion for the initial assessment of the glenohumeral joint. Then, a lateral portal was established as the main viewing portal at the midpoint between the anterior and posterior aspects of the acromion. An anterolateral portal was established as the main working portal during surgical procedure.

The procedure began with an intra-articular evaluation and management of any subscapularis pathology or biceps lesions encountered. After the intra-articular work was completed, the scope was redirected to the subacromial space. In all cases, acromioplasties were also performed to decompress the bony spurs. Biceps tenotomies (*n* = 18; 58% of total 31 patients) were performed if the long head of the biceps tendon was dislocated or torn. The footprint of the tear was prepared using a motorized shaver to eliminate soft-tissue remnants and facilitate bone–tendon healing.

1. Primary repair

A tendon grasper was used to assess the direction of maximum mobility of the tear and, thereby, determine if a tendon end-to-bone repair could be achieved or if the tear had mobile anterior and posterior edges that could be apposed by side-to-side repair. The two medial-row anchors were inserted at the medial border of the footprint of GT adjacent to the articular surface and the lateral anchors were placed on the lateral aspect of the humerus, according to the shape of bridge figuration. The repairs were carried out using the suture bridge technique introduced by Park et al. [17].

2 Patch augmentation

During the operation, if the torn rotator cuff tendon was not sufficiently reducible for primary repair, patch augmentation was carried out as follows: After joint preparation, we reduced torn rotator cuff tendons toward their footprints as much as possible within a tolerable degree of tension, and marked the appropriate points to insert suture anchors. We augmented the supraspinatus muscle and infraspinatus muscle with strands from suture anchors using EXPRESSEW^®^ Flexible Suture Passer. We made all simple ties with the SMC knot technique except the very anterior and very posterior sutures. When augmentation was completed, we measured the size of the defect using an arthroscopic probe to prepare an adequately sized allogenic dermal patch. After the appropriate size was determined, we pulled two strands from the very anterior and very posterior sutures, respectively, then pulled them outside the patient’s shoulder through the accessory anterolateral portal. The other paired strands of each suture were pulled out through the anterior portal for the anterior suture, and through the posterior portal for the posterior suture. Then, using the EXPRESSEW^®^ Flexible Suture Passer, as mentioned above, we passed the two strands anteriorly and posteriorly on the medial side of the prepared allogenic dermal patch. We made a “mega-knot” (mulberry knot) with each strand. Then, we pulled the paired strands that came out through the anterior and posterior portals to place the allogenic dermal patch into the patient’s shoulder through the accessory anterolateral portal. Special care was taken avoid tangling in the strands during this process. After positioning the allogenic dermal patch on top of the patient’s native rotator cuff tendon, we used the suture-bridge technique to fix the lateral side of the allogenic dermal patch. Two or three lateral anchors were inserted, depending on the size of the defect.

#### 2.4.1. Postoperative Protocol

To prevent immediate postoperative failure, a brace immobilizer with an abduction function was applied, with an immobilization period of three to four weeks. Pendulum exercises were started one week post-surgery, followed by active-assisted range of motion (ROM) exercises after 6 weeks. At three months after surgery, the patients were advised to begin isometric muscle exercises using a rubber band. Three months after the operation, patients were allowed to practice light sports activities. A full return to heavy labor or sports was allowed after six months, depending on each patient’s functional recovery.

#### 2.4.2. Statistical Analysis

All statistical analyses were conducted using SPSS (version 27; IBM^®^ SPSS^®^ Statistics). The independent-samples *t*-test and Mann–Whitney test was used to compare the quantitative data between Group A and Group B. Chi-square tests were used to compare the qualitative data of the 2 groups.

The paired *t*-test and Wilcoxon signed rank test was used to compare the difference between preoperative and postoperative scores, and ROM levels, for each group. The statistical significance was set at *p* < 0.05.

## 3. Results

1. Characteristics of Patients

In total, 31 patients who were eligible for the study were enrolled. The demographic data of the two groups did not significantly differ (Table 3).

2 Radiographic Assessment

Radiographic data of the two groups did not significantly differ, except for tear size. Those patients who underwent arthroscopic rotator cuff repair with patch augmentation (Group B) had larger-sized RCTs. The degrees of fat infiltration and global fatty degeneration index (GFDI) values were similar in both groups preoperatively (Table 4). Degrees of re-tears were also similar in both groups postoperatively (Table 5).

3 Functional Assessment

All functional scores improved significantly from baseline for all patients at the last follow-up. In Group A, all functional scores improved, including P-VAS, UCLA, and CSS. Similarly, in Group B, all functional scores improved, and all the scores improved statistically significant. P-VAS scores in Group B were significantly decreased, compared with those in Group A. At last follow-up, significant improvements in ROM values, including FE and ER, were recorded for all patients; however, there was no statistically significant difference in ROM values between the two groups (Table 6).

4 Case

**Figure 2:**
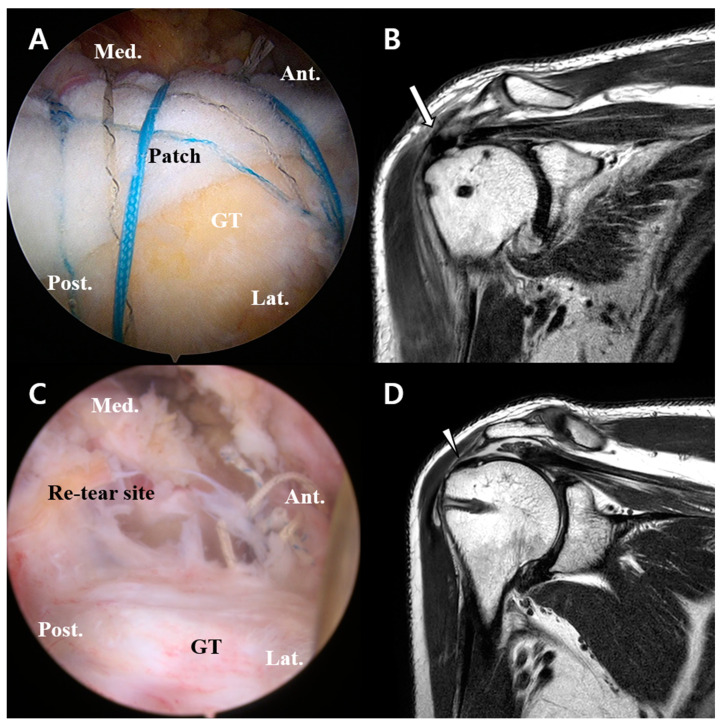
Arthroscopic views and MRIs of re-tear patient. (**A**) Patch augmentation using human allograft for a large rotator cuff tear. (**B**) Postoperative MRI shows a well-maintained patch graft covering GT footprint (arrow). (**C**) Second-look arthroscopic view. A re-tear can be identified, and GT-covering fibrotic tissue can also be seen. (**D**) A T2-weighted coronal image of the right shoulder from a pre-second-operation MRI reveals a major discontinuity (a Sugaya Grade V re-tear) of the supraspinatus tendon. A fibrosis remnant of the allograft patch covers the GT footprint area (arrowhead) despite the re-tear. MRI—magnetic resonance imaging; GT—greater tuberosity.

A 74-year-old male patient underwent revision rotator cuff repair for re-tear. He underwent arthroscopic patch augmentation for a supraspinatus large tear (Figure 2A). On postoperative MRI, the patch graft covered the GT footprint well (Figure 2B). The patient had a P-VAS score of 7 before surgery and a score of 4 at a follow-up about 10 months after surgery. The pain was not severe, but the range of motion of the shoulder was limited, so that further elevation was only possible up to 140 degrees. The limitation of motion was the main symptom, and a revision operation was arranged. A pre-operative MRI for revision rotator cuff repair was performed, which revealed a Sugaya Grade V re-tear of the supraspinatus tendon, in the form of a major discontinuity on the T2-weighted coronal image. The low signal intensity on the GT footprint area was thought to be fibrotic tissue derived from the allograft patch (Figure 2D). This was confirmed with second-look arthroscopy for revision rotator cuff repair (Figure 2C). There was a re-tear of the supraspinatus tendon, but the GT was covered with fibrotic tissue which was thought to be derived from the allograft patch, in line with the MRI evidence. The GT-covering fibrotic tissue could have prevented the occurrence of pain, as we predicted. The patient concerned subsequently underwent arthroscopic rotator cuff (supraspinatus) repair using side-to-side suture technique, and he continues to visit us for follow-up without complaints or surgical complications.

## 4. Discussion

Our study shows that clinical outcomes of patients with re-tear who underwent arthroscopic repair with allograft patch augmentation for large-to-massive RCTs were similar to the outcomes of patients who underwent arthroscopic primary repair, except in the case of P-VAS. The patch augmentation group showed better P-VAS results than the non-augmentation group. Footprint coverage of the GT area is thought to be an important factor affecting the P-VAS.

Compared with traditional rotator cuff primary repair, patch augmentation is known to result in a better healing frequency [18]. Contrarily, in this study, the re-tear rate in the patch augmentation group appeared to be higher. This might have been due to our definition of “re-tear”, which included the Sugaya Grade III classification. The proportions of Sugaya Grade III classifications in our two study groups were 25.0% (3 of 12 patients) in Group A and 36.8% (7 of 19 patients) in Group B, respectively.

In general, regardless of the presence (or absence) of re-tear after rotator cuff repair, good clinical results such as improved shoulder function and reduced pain can be expected with rotator cuff repair, compared with the situation before surgery [18]. This is consistent with the findings of the current study.

The basic objective of the tuberoplasty procedure is to relieve subacromial impingement by reshaping the GT to create a smooth articulation between the GT and the undersurface of the acromion during shoulder abduction [19]. By similar means, subacromial decompression and acromioplasty might also prevent future impingement of the rotator cuff [20]. Acromial morphology is associated with shoulder pathologies and is an important theoretical cause of subacromial impingement, in which the rotator cuff tendons are chronically damaged. Impingement mostly occurs under the anterior and lateral portions of the acromion [21].

Bearing in mind the above, we may say that if the rotator cuff covers the subacromial space by the rotator cuff repair, pain during ROM can be effectively reduced. However, the use of a patch that forms a scaffold with the dermal matrix would result in less tension being applied, compared to a case of repair only with degenerative tissue. So, even if re-tear does occurs, the level of pain is likely to be effectively reduced because GT coverage is sufficient.

Our study had some limitations. First of all, inadequate statistical power was achieved because of the small sample size. We think that a large sample size will be required to obtain sufficient power in future studies. Second, the follow-up period was not the same for all patients. Third, in this study, Sugaya Grade III classifications were included in the re-tear group. Most researchers agree that Sugaya Grades I and II should be considered as no re-tear, and Grades IV and V as re-tear. However, there is uncertainty concerning whether a Sugaya Grade III classification should be regarded as a re-tear or not. The majority of re-tear studies excluded Sugaya Grade III classification from re-tear criteria. However, we considered the Sugaya Grade III partial healing state as a repair failure, in the same way that a partial rotator cuff “tear” refers to an incomplete tear that involves damage to a part of the tendon. However, despite these limitations, we think the results of this study are meaningful because no previous studies compared clinical outcomes in rotator cuff re-tear patients who had either an arthroscopic primary repair or arthroscopic patch augmentation for large-to-massive RCTs.

## 5. Conclusions

Arthroscopic rotator cuff repair with patch augmentation showed promising results in patients with large-to-massive RCTs leading to significant pain reduction, comparing with arthroscopic primary rotator cuff repair. Therefore, when considering arthroscopic repair surgery in patients with large-to-massive RCTs which are sufficiently reducible to the footprint, we recommend that patch augmentation be performed, rather than primary repair, on account of the better clinical results achieved by patch augmentation in terms of pain, even if re-tear subsequently occurs.

## Figures and Tables

**Table 1:** Goutallier fatty degeneration classification system.

Grade	Muscle Description
Grade 0	Completely normal muscle, without any fatty streaks
Grade I	Muscle contains some fatty streaks
Grade II	Fatty infiltration is important, but there is more muscle than fat (<50% involvement)
Grade III	Equal amounts of fat and muscle (50% involvement)
Grade IV	More fat than muscle is present (>50% involvement)

**Table 2:** Sugaya classification system.

Grade	Definition
Grade I	Sufficient thickness with homogenous low intensity
Grade II	Sufficient thickness with partial high intensity
Grade III	Insufficient thickness without discontinuity
Grade IV	Presence of a minor discontinuity
Grade V	Presence of a major discontinuity

**Table 3:** Demographic data of the patients.

Variable	Group A (*n* = 12)	Group B (*n* = 19)	*p* Value
Age (yr) Follow up periods (yr)	64.7 ± 9.4 (44–77) 4.08 ± 0.73	64.8 ± 5.7 (55–78) 3.88 ± 0.77	0.948 ^a^ 0.842 ^a^
Sex			0.687 ^b^
Male	8	14	
Female	4	5	
Duration of symptoms (Months)	10.4 ± 18.4	11.0 ± 26.8	0.943 ^a^
Smoking?			0.745 ^b^
Yes	1	1	
No	11	18	
Previous traumatic event?			0.89 ^b^
Yes	6	10	
No	6	9	

Note—Except where noted otherwise, data are number of patients. Data are presented as mean ± standard deviation. ^a^ independent *t*-test and Mann–Whitney test, ^b^ chi-square and Fisher’s exact test.

**Table 4:** Comparison of preoperative radiographic findings between the two groups.

	Group A (*n* = 12)	Group B (*n* = 19)	*p* Value
Tear size (mm)	33.1 ± 3.5	39.7 ± 7.3	**0.002** ^a^
Large (≥3 and <5 cm)	12	17	
Massive (≥5 cm)	0	2	
Degree of fat infiltration			
Supraspinatus			0.740 ^b^
Grade I	3	3	
Grade II	2	5	
Grade III and IV	7	11	
Subscapularis			0.474 ^b^
Grade I	8	13	
Grade II	1	3	
Grade III and IV	3	3	
Infraspinatus			0.565 ^b^
Grade I	7	12	
Grade II	3	5	
Grade III and IV	2	2	
GFDI (Result value)	2.0 ± 0.7	1.8 ± 0.6	0.410 ^c^

Note—Except where noted otherwise, data are number of patients. Data are presented as mean ± standard deviation. Values shown in bold type are statistically significant. The Goutallier classification system was used. GFDI—global fatty degeneration index. ^a^ chi-square, ^b^ Fisher’s exact test, ^c^ independent *t*-test.

**Table 5:** Comparison of postoperative radiographic findings between the two groups.

	Group A (*n* = 12)	Group B (*n* = 19)	*p* Value
Degree of re-tear			0.729 ^a^
Grade III	3	7	
Grade IV	4	4	
Grade V	5	8	

Note—Except where noted otherwise, data are number of patients. The Sugaya classification system was used for re-tear degree assessment. ^a^ Fisher’s exact test.

**Table 6:** Comparison of functional outcomes between the two groups.

	Preoperative	Postoperative	*p* Value	
			Differences between Preoperative and Postoperative	Increments between the Two Groups
Score				
P-VAS			**0.000** ^a^	**0.000** ^b^
Group A	6.3 ± 1.5	4.5 ± 1.4	**0.000** ^a^	
Group B	5.2 ± 1.9	1.5 ± 0.9	**0.000** ^a^	
SST			**0.000** ^a^	0.618 ^b^
Group A	4.5 ± 2.1	6.6 ± 2.9	0.062 ^a^	
Group B	4.7 ± 1.8	7.4 ± 1.6	**0.000** ^a^	
UCLA			**0.000** ^a^	0.142 ^b^
Group A	15.8 ± 7.3	21.3 ± 6.1	**0.037** ^a^	
Group B	13.1 ± 42	22.6 ± 6.9	**0.000** ^a^	
ASES			**0.000** ^a^	0.164 ^b^
Group A	57.1 ± 18.7	66.8 ± 20.1	**0.004** ^a^	
Group B	59.6 ± 14.1	73.6 ± 16.9	**0.000** ^a^	
CSS			**0.000** ^a^	0.264 ^b^
Group A	40.6 ± 20.8	62.8 ± 17.6	**0.001** ^a^	
Group B	44.1 ± 16.0	60.2 ± 11.3	**0.000** ^a^	
Range of Motion				
FE, degrees			**0.021** ^a^	0.337 ^b^
Group A	136.7 ± 66.7	172.5 ± 23.0	0.120 ^a^	
Group B	160.0 ± 37.1	173.7 ± 20.9	0.050 ^a^	
ER, degrees			**0.009** ^a^	0.124 ^b^
Group A	46.7 ± 22.1	63.3 ± 16.0	**0.012** ^a^	
Group B	70.0 ± 25.8	75.5 ± 18.4	0.223 ^a^	
IR, spine level			0.826 ^a^	0.536 ^b^
Group A	6.7 ± 5.7	5.3 ± 6.4	0.580 ^a^	
Group B	5.4 ± 4.7	5.8 ± 4.9	0.819 ^a^	

Note—Except where noted otherwise, data are number of patients. Data are presented as mean ± standard deviation. Values shown in bold type are statistically significant. P-VAS—Pain Visual Analog Scale; SST—Simple Shoulder Test; UCLA—University of California Los Angeles; ASES—American Shoulder and Elbow Surgeons; CSS—Constant Shoulder Score.; FE—forward elevation; ER—external rotation; IR—internal rotation. ^a^ paired *t*-test, ^b^ independent *t*-test and Mann–Whitney test.

## Data Availability

Not applicable.
